# Electronic Structure of Biexcitons in Metal Halide Perovskite Nanoplatelets

**DOI:** 10.1021/acs.jpclett.4c01719

**Published:** 2024-07-12

**Authors:** Juan I. Climente, José L. Movilla, Josep Planelles

**Affiliations:** † Departament de Química Física i Analítica, 16748 Universitat Jaume I, E-12080, Castelló de la Plana, Spain; ‡ Dept. d’Educació i Didàctiques Específiques, 16748 Universitat Jaume I, 12080, Castelló, Spain

## Abstract

A theoretical description of biexcitons in metal halide perovskite nanoplatelets is presented. The description is based on a variational effective mass model, including polaronic effects by means of a Haken potential. The strong quantum and dielectric confinements are shown to squeeze the biexciton under the polaronic radius, which greatly enhances Coulomb attractions and (to a lesser extent) repulsions. This explains the need for effective dielectric constants approaching the high-frequency limit in previous simulations, and the binding energies exceeding 40 meV observed in single-monolayer nanoplatelets. Biexcitons are formed by a pair of weakly interacting excitons, with a roughly rectangular geometry. This translates into a constant ratio between biexciton and exciton binding energies (2D Haynes rule) well below the ideal value of Δ_BX_/Δ_X_ = 0.228 proposed for squared biexcitons. The ratio is independent of the number of monolayers in the platelet, but it does depend on the lateral and dielectric confinement.

## Graphical abstract

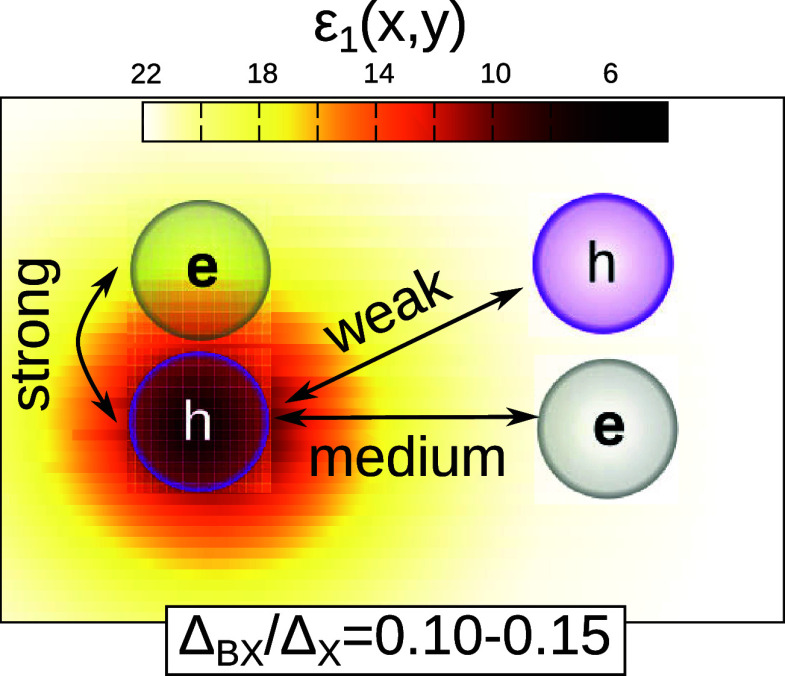

At present, quasi-2D lead halide perovskites—nanoplatelets (NPLs) and layered halide perovskites (LHPs)—are among the most promising colloidal materials for the development of improved photovoltaic and optoelectronic devices. The success of these systems arises from the combination of natural advantages of 3D lead halide perovskites (ease of synthesis, compositional tunability, high optical efficiency and long carrier diffusion lengths) with a greater stability and directional emission, imparted by the presence of coordinating ligands.^1–5^ Equally important is the fact that the limited thickness of these structures (down to a single monolayer of PbX_4_^2–^ octahedra) introduces two new degrees of freedom in the design of the electronic structure, namely quantum and dielectric confinement.^6–8^ Quantum confinement increases the band gap energy with respect to bulk values, which is seen as a blueshift of emission and absorption band edges (up to 0.6 eV for layered hybrid organic–inorganic perovskites). In turn, dielectric confinement enhances Coulomb interactions of photoinduced carriers in the inorganic crystal. This is reflected by excitonic binding energies over 200 meV, 1 order of magnitude greater than those of bulk and large nanocrystals. An additional extension of the band gap is also provided by self-energy effects,^6^ which are inherent to dielectric confinement.^9^

The influence of quantum and dielectric confinement on the exciton (X) electronic structure of quasi-2D metal halide perovskites is akin to that previously reported in group II–VI colloidal NPLs.^10–13^ An important differential trait between such materials, however, is the softness and polarizability of the perovskite lattice. The latter contains not only Pb but also large cations (typically Cs, methylammonium or formamidinium), which results in a strong X-phonon (polaron) coupling.^14–16^ Several nontrivial effects have been associated with polaronic coupling in halide perovskites. These include exciton self-trapping,^17–19^ reduced mass renormalization,^20,21^ complex spectral structure,^16,22–24^ enhanced intra-X^25^ and inter-X^26^ interaction.

Exciton binding energies and recombination rates in metal halide perovskites (bulk and nanocrystals) have been successfully interpreted by means of simple—yet intuitive—effective mass models.^21,25,27,28^ A more demanding case is that of quasi-2D NPLs, where polaronic effects coexist with pronounced quantum and dielectric confinement. To deal with this system, we recently developed an effective mass model combining all three physical factors.^29^ Polaronic effects were described using a Haken potential, which discriminates the different lattice screening at short distances (electronic screening) and at long ones (electronic plus ionic screening).^30–32^ Dielectric confinement effects, in turn, were described with an image charge formalism, for both long-range (Coulomb) and short-range (Yukawa) terms of the Haken potential. The calculated binding energies were in remarkable agreement with experimental measurements of Ruddlesden–Popper perovskites [(PEA)_2_(MA)_*n*−1_Pb_*n*_I_3*n*+1_, with *n* = 1, 2, 3, PEA = phenylethylammonium, MA = methlyammonium],^33^ and the self-energy with that obtained from ab initio DFT calculations.^34^

In the present work, we extend our study to the case of biexcitons (BXs). We analyze the dependence of the BX binding energy and wave function on the system thickness, dielectric environment and lateral dimensions. Our model is based on a variational Quantum Monte Carlo—effective mass method, similar to that we used to investigate BXs in CdSe NPLs,^35^ albeit incorporating polaronic effects through a Haken potential. The variational nature of the study allows us to rationalize a number of experimental and theoretical observations of these systems in simple terms. It is also useful to assess on the validity of the 2D Haynes rule, which is occasionally used in the literature to establish a relation between BX and X binding energies.^36,37^ Furthermore, lateral confinement of NPLs is shown to be a relevant factor in manipulating the electronic structure. The detailed understanding of the BX electronic structure we provide contributes to the ongoing endeavor to design BX properties in quasi-2D halide perovskites,^36–45^ aiming at lasing applications, among others. Specifically, control of Δ_BX_ is needed to ensure high operation temperatures^40,43^ and low thresholds of amplified spontaneous emission,^46^ while knowledge of the wave function is relevant to anticipate Auger recombination,^47,48^ X–X annihilation,^49–51^ and radiative recombination^35^ rates.

We calculate the BX ground state energy and wave function from a *k*·*p* Hamiltonian for two uncoupled (conduction and valence) bands:

$$H_{\mathrm{BX}}=\underset{i=e_{1},e_{2},h_{a},h_{b}}{\sum }\left(\frac{{\mathrm{p̂}}^{2}}{2m_{i}}+V_{i}\right)+V_{c}\left(r_{e_{1}},r_{e_{2}}\right)+V_{c}\left(r_{h_{a}},r_{h_{b}}\right)+\underset{i=e_{1},e_{2}}{\sum }\underset{j=h_{a},h_{b}}{\sum }V_{c}\left(r_{i},r_{j}\right)+2E_{\mathrm{gap}}$$ 1

where *m* is the effective mass, **p̂** the momentum operator, and *E*_gap_ the bulk band gap of the perovskite crystal. The single particle potential is *V*_*i*_ = *V*_*i*_^conf^ + *V*_*i*_^self^. Here, *V*_*i*_^conf^ is the 3D confining potential, which is zero inside the cuboidal NPL with dimensions *L*_*x*_ × *L*_*y*_ × *L*_*z*_ (see Figure 1a), and infinite outside. *V*_*i*_^self^ is the self-energy potential. *V*_*c*_(**r**_*i*_, **r**_*j*_) terms represent the Coulomb interaction between carriers. Both *V*_*i*_^self^ and *V*_*c*_(**r**_*i*_, **r**_*j*_) account for dielectric confinement by using quantum well image charges, with inclusion of long- and short-range interactions. Detailed expressions can be found in ref 29. These are based on the Haken potential whose expression in bulk—before including quantum and dielectric confinement—we show next for clarity of exposition. In atomic units, the potential exerted by a source charge *i* on a test charge *j* reads as follows:^30^

$$V_{ij}^{\mathrm{bulk}}\left(r\right)=\frac{q_{i}q_{j}}{\epsilon_{s}r}+\frac{q_{i}q_{j}}{\epsilon^{*}r}\left(\frac{e^{-\beta_{i}r}+e^{-\beta_{j}r}}{2}\right)$$ 2

Here, *r* is the distance between charges, *q* the elementary charge (positive or negative), ϵ_*s*_ the static dielectric constant and ϵ_∞_ the high frequency (optical) one. The term $\left(\frac{1}{\epsilon_{\infty }}-\frac{1}{\epsilon_{s}}\right)$ = $\frac{1}{\epsilon^{*}}$ represents the ionic screening of carriers. β_*i*_ is the inverse of the electron and hole polaron radius: β_*i*_ = *l*_*i*_^–1^, which is related to the longitudinal optical phonon frequency. One should note that the first term is a Coulomb interaction with full dielectric screening (electronic plus ionic, ϵ_*s*_), which prevails at long distances, *r* ≫ *l*_*i*_. The second term is a short-range (Yukawa) one, which starts prevailing at distances *r* ≲ *l*_*i*_, where the ionic contribution to dielectric screening is lost (ϵ_∞_ dominates). Equation 2 constitutes a simple model of X-polaron interactions, which has been shown to improve estimates of X binding energies in different semiconductors,^30–32^ including lead halide perovskites.^21,25,27,29^

**Figure 1:**
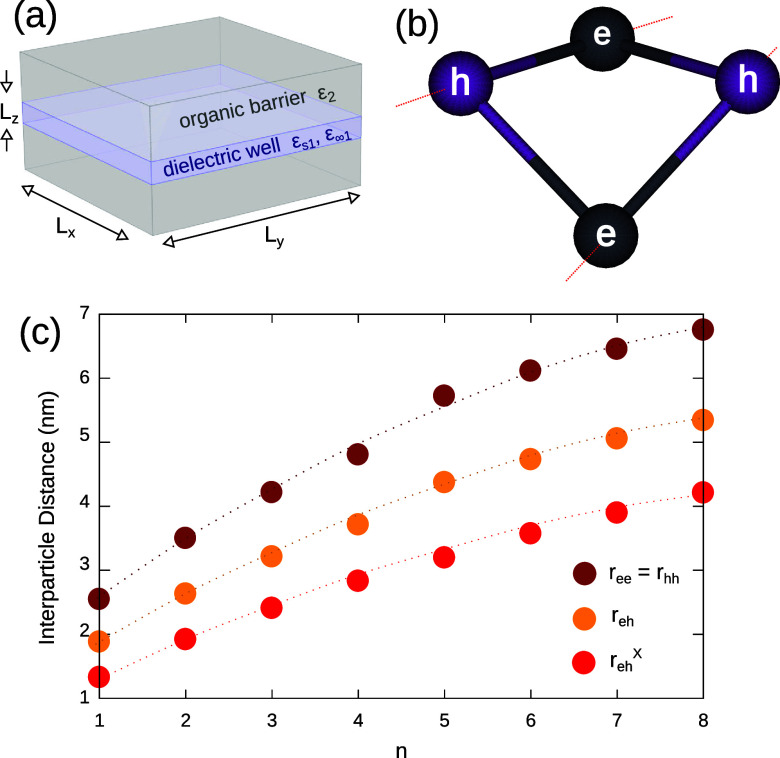
(a) Schematic of the system under study. (b) Average geometry of a BX in a MAPbI_3_ NPL with *n* = 2. Gray and purple spheres represent the most probable location of electrons and holes, respectively. A nonplanar, distorted square is formed. (c) Size dependence of BX and X internal distances on the number of layers *n*. Dots are calculated values, lines are guide to the eyes.

The Hamiltonian described by eq 1 is solved with a four-parameter variational wave function for the BX ground state:

3 $$\Psi_{\mathrm{BX}}\left(r_{e_{1}},r_{e_{2}},r_{h_{a}},r_{h_{b}}\right)=N_{\mathrm{BX}}\Phi_{e}\left(r_{e_{1}}\right)\Phi_{e}\left(r_{e_{2}}\right)\Phi_{h}\left(r_{h_{a}}\right)\Phi_{h}\left(r_{h_{b}}\right)F\left(r_{1a},r_{1b},r_{2a},r_{2b},r_{12},r_{ab}\right)$$

Here, *N*_BX_ is the normalization factor, Φ_e_ and Φ_*h*_ are the noninteracting electron and hole envelope functions, Φ_*i*_ = ∏_α=*x*, *y*, *z*_ cos­(πα/*L*_α_). *F* is the correlation factor, described by

$$F\left(r_{1a},r_{1b},r_{2a},r_{2b},r_{12},r_{ab}\right)=e^{-Z\left(s_{1}+s_{2}\right)/2}\;\cosh\left(ZQ\frac{t_{1}-t_{2}}{2}\right)\times e^{Z\beta r_{12}/\left(1+Z\alpha r_{12}\right)}\;e^{Z\beta r_{ab}/1+Z\alpha r_{ab}}$$ 4

where *r*_12_, *r*_*ab*_, *r*_1*a*_, *r*_1*b*_, *r*_2*a*_, *r*_2*b*_ are *in-plane* interparticle distances, *s*_1_ = *r*_1*a*_ + *r*_1*b*_, *s*_2_ = *r*_2*a*_ + *r*_2*b*_, *t*_1_ = *r*_1*a*_ – *r*_1*b*_, and *t*_2_ = *r*_2*a*_ – *r*_2*b*_. The variational parameters to optimize are *Z*, *Q*, β, and α.

Ψ_BX_ is reminiscent of the trial wave function used by Hylleraas and Ore for BXs in bulk.^52^ Thus, the first term in eq 4 describes Slater correlation factors, analogous to those used to account for carrier attraction of individual Xs.^13,28^ *Z* can be seen as a scaling factor reflecting the strength of electron–hole attraction. *Q* allows for a nonsymmetric interaction in the BX. In bulk, *Q* = 0 corresponds to a perfectly bound (squared) BX, while *Q* = 1 corresponds to a pair of dissociated Xs. The last two exponentials in eq 4, involving α and β parameters, are missing in Hylleraas’ wave function. They are Padé–Jastrow factors representing the carrier repulsion correlation.

A straight variational solution of Ψ_BX_ is not viable because of the large number of dimensions. We then resort to a Quantum Monte Carlo integration, as described in ref 35. For the materials and geometries we consider, the repulsive correlation terms are found to be minor contributions. Thus, we fix α = β = 1 and simply scan the space of parameters (*Z*, *Q*). The number of steps and walkers in the Monte Carlo integration is chosen to give accuracy of ∼1 meV. Exciton energies and wave functions, when needed, are calculated following ref 29. It is worth noting that electron–hole exchange interaction, which has proved significant in thin perovskite NPLs,^53^ may reduce the symmetry of the Bloch functions and hence the degeneracy of the exciton ground-state multiplet, but it does not affect the envelope part of the wave function. It then follows that eq 3 and the binding energies we obtain remain unaffected.

To investigate the connection between polaronic effects, quantum confinement and dielectric confinement, we model NPLs made of MAPbI_3_, a prototypical hybrid metal halide perovskite.^1,2^ Material parameters are taken from ref 29. Specifically, dielectric constants are ϵ_*s*1_ = 22.0 and ϵ_∞1_ = 5.6,^34^ effective masses *m*_*e*_ = 0.19 and *m*_*h*_ = 0.22. Polaron radii of *l*_*e*_ = 1/β_*e*_ = 0.94 nm and *l*_*h*_ = 1/β_*h*_ = 1.01 nm are used. These values successfully reproduced the experimental exciton binding energies of (PEA)_2_(MA)_*n*−1_Pb_*n*_I_3*n*+1_ layers, with *n* = 1, 2, 3.^54^ The low polarizability of the organic environment is characterized by ϵ_2_ = 2.^55^ Unless otherwise stated, we take lateral dimensions *L*_*x*_ = *L*_*y*_ = 30 nm and vary the NPL thickness as *L*_*z*_ = *na*, where *n* is the number of PbI_4_^–2^ octahedra (layers) and *a* = 0.63 nm their lattice parameter.^14^ The results we obtain are then representative of NPLs in the weak lateral confinement regime. They are also a good description of LHPs where interlayer spacing is not very short, as interactions between layers are then weak and a composite picture of the system as independent layers hold.^7,29,38^

It is convenient to start the study by analyzing the *average* geometry of the BX in NPLs. We calculate the most probable location of electrons and holes from the internal coordinates recorded during the Monte Carlo random walks, using Metropolis algorithm estimators.^56,57^ Figure 1b shows the resulting BX for a NPL with *n* = 2 layers. Gray and purple spheres stand for electrons (*e*) and holes (*h*), respectively. The molecular geometry is a distorted square, similar to that taking place in CdSe NPLs,^35^ with *ehe* angles of 83.1° and a dihedral angle of 124.7°. This distribution of charges is close to the ideal squared distribution expected from electrostatics, where BX attractions are maximized by keeping particles of opposite sign along the edges of a planar square, and repulsions minimized by keeping particles of the same sign across the diagonal.^58^ However, deviations from this limit are present, and we shall see that they have profound implications on the BX binding energy. The main reason is that both *m*_*e*_ and *m*_*h*_ are small. Together with the confinement, this provides the BX with a strong kinetic energy, which counteracts electrostatic effects. A first manifestation is the lack of planar symmetry of the BX. When both holes are aligned on an *z* plane, electrons have the maximum density probability on such a plane, but the density decreases only gradually away from it. Because the volume of integration increases with the distance, the maximum probability takes place outside the plane.

Figure 1c shows the average distances between particles within the BXs as a function of the NPL thickness. One can see that the distances shrink when the number of constituent layers *n* decreases, for both electron–hole (*r*_*eh*_, orange dots) and electron–electron or hole–hole (*r*_*ee*_ = *r*_*hh*_, brown dots) distances. The same phenomenon is observed for single excitons (*r*_*eh*_^X^, red dots), which are systematically smaller. The squeezing of the wave function with increasing confinement is a consequence of the enhancement of Coulomb interactions when the system goes 2D. For excitons in halide perovskite crystals, it has been observed experimentally^33^ and theoretically.^29^ We confirm the same trend in BXs, despite the presence of repulsions.

Having seen BX size dependence on the NPL thickness, we are in the condition to study the binding energy dependence. We follow the usual definition for bulk: Δ_BX_ = 2*E*_X_ – *E*_BX_, with *E*_X_ the exciton ground-state energy and *E*_BX_ the biexciton one. The convenience of this definition in confined structures is that it closely corresponds to the spectroscopical shift between X and BX emission resonances.^45^ Solid dots in Figure 2a show the calculated Δ_BX_ with full inclusion of effects (quantum and dielectric confinement, polaronic effects through Haken’s potential). The binding energy is positive (i.e., BXs are bound), and increases as the NPL becomes thinner, which is consistent with the BX shrinking observed in Figure 1c. For *n* = 1 (monolayered NPL), Δ_BX_ reaches 42 meV. This value matches the experimentally measured Δ_BX_ for such systems, which ranges between 40 and 55 meV.^40–42,45^ It is also close to the 44 meV calculated by Cho and co-workers using Diffusion Quantum Monte Carlo.^36^ The overall agreement supports the validity of our simpler variational method in the most demanding (quasi-2D) situation. For more quantitative estimates of Δ_BX_, one should bear in mind that optical phonon modes and the strength of their coupling to electronic charges may depend on the size of the perovskite nanostructure.^59,60^ Likewise, temperature affects the dynamic lattice disorder.^42^ These effects may vary the polaron radius from our guess (*l*_*e*_ ≈ *l*_*h*_ ≈ 1 nm). In the Supporting Information, we show how Δ_BX_ depends on the polaron radius. Larger polaron radii imply greater BX stability. For *l*_*e*_ ≈ *l*_*h*_ ≥ 2 nm, Δ_BX_ ≈ 55 meV is obtained, which is close to the upperbound experimental values reported.^42^

**Figure 2:**
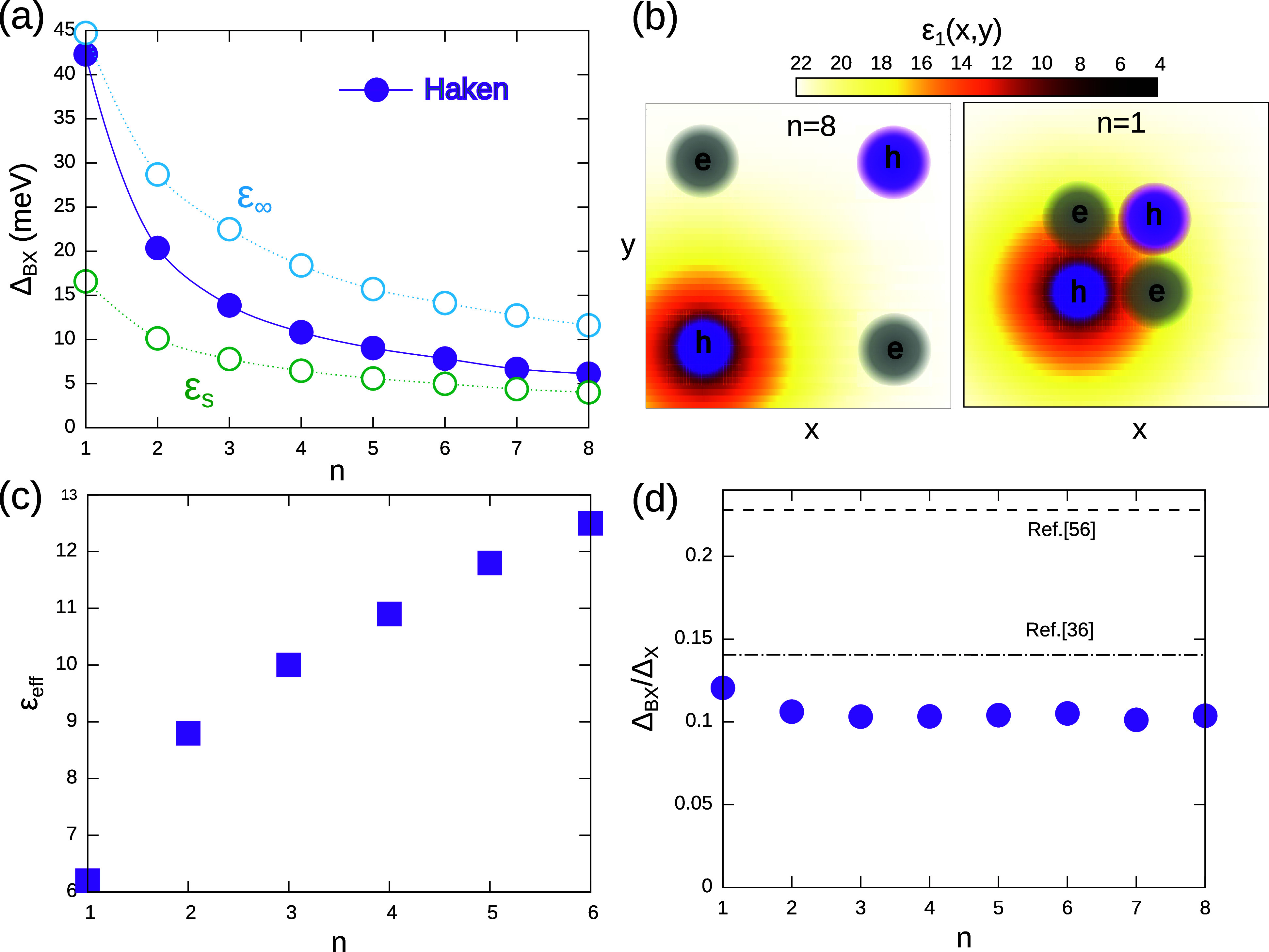
(a) BX binding energy as a function of the NPL thickness. Solid dots are calculated points with Haken’s potential. Green and blue circles are calculated values in the limits of static and high-frequency dielectric screening. (b) Sketch of the BX squeezing in the plane of the NPL as the thickness decreases. The left, bottom hole is considered to be the source charge, inducing position-dependent lattice polarization. When charges get closer, they feel weaker screening. This effect is more important for electron–hole pairs than for carriers of the same sign, which results in a greater enhancement of attractions over repulsions. (c) Effective dielectric constant of the MAPbI_3_ NPL for different number of layers, *n*. (d) Ratio of BX-to-X binding energy. It is roughly constant against the NPL thickness (Haynes rule in 2D), but the value is well below that anticipated for squared BXs (dashed line), and closer to recent numerical estimates (dot-dashed line).

It is worth noting that the computational simulations in ref 36 assumed a dielectric constant of ϵ_1_ = 6.1 for the inorganic layer, namely, the high-frequency constant of pure PbI_2_ crystals.^61^ The convenience of using ϵ_∞_ rather than ϵ_*s*_ was based on experimental evidence that Δ_*X*_ ≫ ℏω_*LO*_ in layered perovskites with small *n*.^38^ One then expects electron–hole distance to be under the polaron radius, so that ϵ_∞_ prevails in eq 2. However, it is not clear that the same holds for Δ_BX_, which is of the same order as ℏω_LO_. In our model, by contrast, we obtain agreement with experiments directly from ϵ_*s*_ and ϵ_∞_ of bulk MAPbI_3_. It is the interplay between confinement and polaronic effects (through the Haken potential) that determines which is the actual screening of charges felt by the BX. To make this point clear, in Figure 2a, we compare our calculations with those in the limit of Coulomb interaction with static (green circles) and dynamic (blue circles) screening. For bulky systems, Δ_BX_ is close to the static limit, but it approaches the dynamic one for thin NPLs. A similar behavior has been reported for X in bulk^25^ and quasi-2D^29^ halide perovskites. The explanation is as follows: with decreasing *n*, Ψ_BX_ shrinks and a greater amount of charge density lies in the vicinity of the polaronic radius of the other carriers (*l*_*e*_, *l*_*h*_). In that region, the ionic contribution of the screening is gradually lost. As a result, the charges start feeling the dynamic (rather than static) dielectric constant. The process is qualitatively depicted in Figure 2b. In the figure we consider one of the holes to be the source charge, and map the space-dependent dielectric constant it builds on the NPL plane. For *n* = 8 NPLs (left panel), the other (test) charges are far and feel ϵ_1_ ≈ 22, but for *n* = 1 (right panel) the charges become tightly packed and benefit from a reduced ϵ_1_. Interestingly, because *r*_*eh*_ < *r*_*ee*_ = *r*_*hh*_, attractions benefit more from dynamic screening than repulsions (see the *n* = 1 case in Figure 2b). In other words, in BX short distance polaronic effects entail a greater enhancement of attractions than of repulsions. This helps to explain the large Δ_BX_ observed in experiments.

To establish a connection between Haken’s potential and the usual Coulomb potentials used in earlier effective mass models, in Figure 2c, we show the *effective* dielectric constant which reproduces Δ_BX_ calculated with the Hamiltonian described by (Eq. 1), but using a Coulomb term with a fixed constant inside the NPL, ϵ_1_ = ϵ_eff_, which does not depend on the interparticle distance. The value of ϵ_eff_ is clearly dependent on the NPL thickness. It evolves toward the dynamic limit as *n* decreases, in line with the expectations from Figure 2(a). For monolayer NPLs, we obtain ϵ_eff_ = 6.2, which explains the value that had to be assumed in ref.^36^

The ratio of BX-to-X binding energies, Δ_BX_/Δ_X_, has been a matter of debate for different quasi-2D materials.^35,36,58,62,63^ The fundamental question is whether a linear scaling holds, such that the stronger the X binding, the stronger the BX one. The existence of a constant ratio, independent of the thickness, is sometimes known as the 2D Haynes rule. Early theoretical studies for epitaxial quantum wells proposed Δ_BX_/Δ_X_ = 0.228, a universal value which does not depend on the material or thickness, as long as the BX is well described as a planar square.^58^ This value has been recently used in experimental works of LHPs.^37^ However, the question remains of how finite thickness, dielectric confinement, and polaronic effects affect Δ_BX_/Δ_X_. None of these effects was accounted for in ref 58, and we have already seen in Figure 1b that they introduce deviations from a planar square geometry. In fact, numerical studies in LHP suggest a ratio of 0.14 instead.^36^ We calculate Δ_BX_/Δ_*X*_ for different NPL thicknesses. The results, shown in Figure 2d, confirm that a 2D Haynes rule holds, but the ratio is well below the universal value of ref 58 and closer to that obtained with Diffusion Quantum Monte Carlo.^36^

The variational nature of our model allows us to shed light on the origin of the low value of Δ_BX_/Δ_X_ in metal halide NPLs. As mentioned when describing Ψ_BX_ in eq 3, in the regime of weak lateral confinement, *Q* relates to the degree of BX molecular coupling. *Q* = 0 corresponds to a perfectly bound BX, *Q* = 1 to two independent X. A squared BX^58^ implicitly assumes *Q* = 0. In Figure 3(a) we plot the full dissociation curve for the BX in a NPL with *n* = 1. One can see that the minimum is not found in the *Q* = 0 limit, but rather at intermediate values. This means the BX is formed by a pair of Xs, with intra-X interactions being stronger than inter-X ones. This is the point of balance between electrostatic energy (favoring *Q* = 0) and kinetic energy (favoring *Q* = 1).

**Figure 3:**
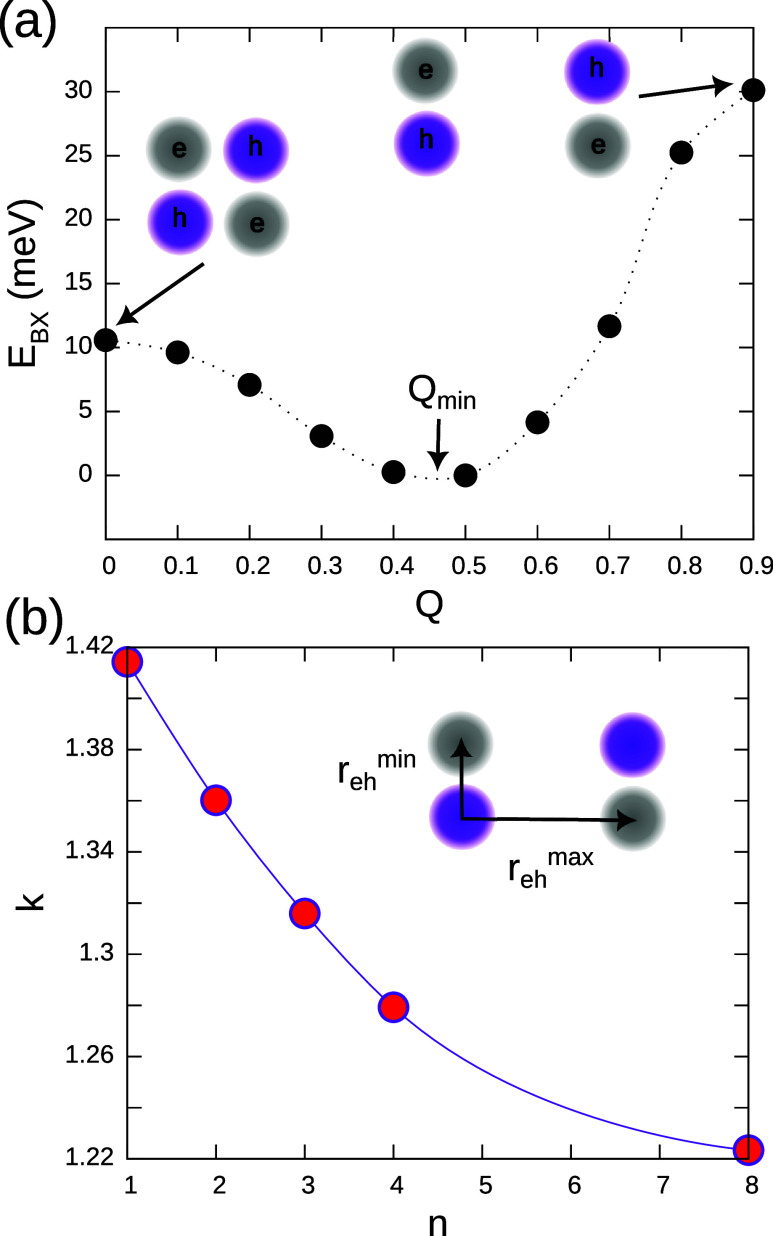
(a) Dissociation curve of a BX in a monolayered MAPbI_3_ NPL (*n* = 1). *Q* is a (dimensionless) variational parameter (see eq 3). The minimum energy is found at *Q*_min_ ≠ 0, suggesting the BX is formed by weakly interacting X, rather than a perfect (squared) molecule. (b) Ratio of the larger and smaller electron–hole distance within the BX, for NPLs with different thickness. *k* = 1 means squared BX. *k* > 1 is indicative of a rectangular charge distribution.

Closer insight into the actual BX geometry is gained by realizing that the charge distributions displayed in Figures 1b and 1c correspond to average values. The fact that *Q*_min_ ≠ 0 is indicative that the BX is really formed by a pair of weakly interacting Xs, with a rather rectangular shape. Upon averaging over different rectangular configurations (considering the permutation symmetry of e_1_– e_2_, and *h*_*a*_ – *h*_*b*_), one obtains a seemingly squared distribution. To quantify the rectangular nature of BXs, we recalculate the interparticle distances avoiding permutation symmetry. For every step in the random walk, we choose a hole and estimate separately the distance with the closer electron and that with the more distant one. Electron–hole distances are then defined as

5 $$r_{eh}^{\min}=\frac{1}{2}\left[\min\left(r_{a1},r_{a2}\right)+\min\left(r_{b1},r_{b2}\right)\right]$$

6 $$r_{eh}^{\max}=\frac{1}{2}\left[\max\left(r_{a1},r_{a2}\right)+\max\left(r_{b1},r_{b2}\right)\right]$$

Even in the absence of excitonic correlations, this definition leads to *r*_*eh*_^max^/*r*_*eh*_^min^ > 1, because there is always one particle farther than the other. To reduce this bias, we renormalize using the electron–hole distances of uncorrelated BX. The latter is easily calculated setting *Z* = 0 in Ψ_XX_. The coefficient

7 $$k=\frac{\left(r_{eh}^{\max}/r_{eh}^{\min}\right)}{{\left(r_{eh}^{\max}/r_{eh}^{\min}\right)}_{Z=0}}$$

gives a good approximation on how carrier–carrier interactions affect the BX shape. *k* = 1 corresponds to a squared geometry, and *k* > 1 to a rectangular one, with larger inter-X than intra-X distances. Figure 3b shows that correlations make the BX rectangular (*k* > 1) in all instances. The thinner the NPL, the more pronounced the departure from the square limit.

While the NPL thickness is a major control parameter, BX properties can also be manipulated by varying the dielectric environment and the lateral confinement. The former is achieved by using organic ligands of different polarizability to passivate the NPL.^8^ The latter, by growing NPLs with lateral sizes introducing additional quantum confinement. Moderate control of the lateral sizes down to ∼10 nm has been demonstrated.^50,64^

In Figure 4, we show that both parameters can be exploited to vary Δ_BX_ in a wide range. In a monolayered NPL (*n* = 1), the dielectric constant of the environment modulates Δ_BX_ from 23 to 48 meV, as ϵ_2_ changes from ϵ_∞1_ = 5.6 (reduced mismatch) to ϵ_2_ = 1 (NPL surrounded by vacuum), see Figure 4a. A similar enhancement (of ∼100%) can be obtained through lateral confinement. To illustratre this claim, we choose a NPL with *n* = 2 and vary the side *L* = *L*_*x*_ = *L*_*y*_ (see Figure 4b). In this case, Δ_BX_ goes from 20 to 42 meV as one changes from nonconfined (*L* = 30 nm) to moderately confined (*L* = 7.5 nm) NPLs. It is interesting to note that for a NPL with *n* = 2 and *L* = 7.5 nm, Δ_BX_ = 42 meV is as large as in a *n* = 1 NPL with large area (Figure 2a). This implies that lateral confinement, despite having a minor contribution on the X energy, can have a significant influence on the BX binding strength. This result is consistent with the strongly bound biexcitons (Δ_BX_ = 74 meV), reported for very narrow (rodlike) CsPbBr_3_ NPLs.^37^

**Figure 4:**
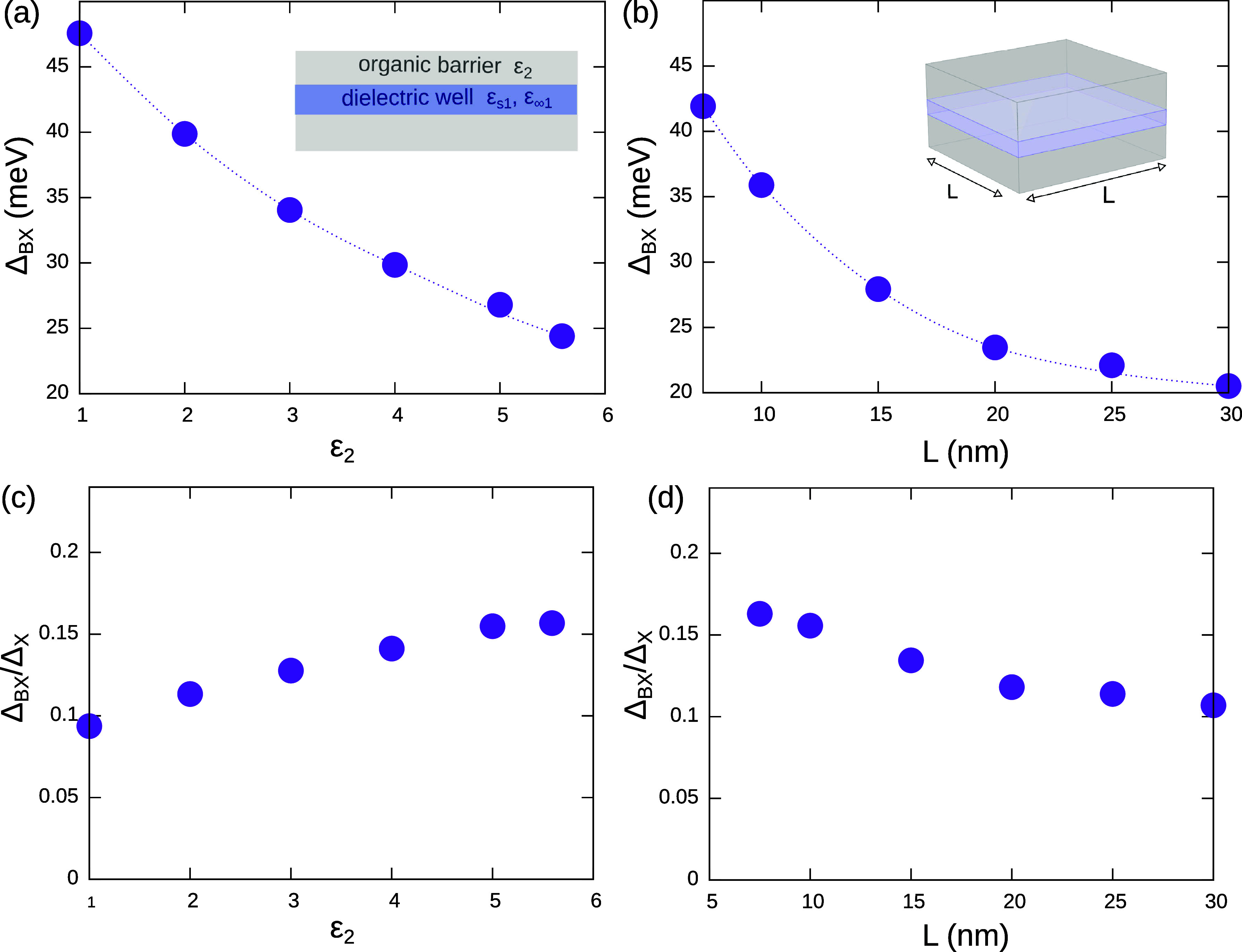
(a) Effect of the dielectric confinement exerted by the outer medium on a *n* = 1 MAPbI_3_ NPL. (b) Effect of lateral confinement in a NPL with thickness *n* = 2 and *L*_*x*_ = *L*_*y*_ = *L*. (c and d) Corresponding calculations of the BX-to-X binding energy ratio (Haynes rule). Both types of confinement have a significant impact on the BX properties. The insets are schematics of the structure under study.

We argued in Figure 2d that Δ_BX_/Δ_X_ is roughly independent of the NPL thickness (2D Haynes rule). The actual value of such a ratio is however dependent on other structural and compositional parameters. It has been shown that it depends on the electron and hole masses.^62^ In Figures 4c and 4d, we go further and show that it depends on the dielectric and lateral confinement as well. In a thin MAPbI_3_ NPL, Δ_BX_/Δ_X_ decreases from ∼0.15 to ∼0.10 as the dielectric confinement becomes more severe. That is, intra-X interactions benefit more than inter-X ones from the enhanced Coulomb coupling. Lateral confinement works conversely. Δ_BX_/Δ_X_ increases when the confinement becomes stronger. This implies that reducing the NPL area stimulates inter-X interactions more than intra-X ones, which is consistent with the larger radius of BX observed in Figure 1c.

In conclusion, the electronic structure of the BX ground state in metal halide NPLs is defined by the interplay between quantum confinement, dielectric confinement, and polaronic effects. By means of an effective mass-variational Quantum Monte Carlo model, which accounts for all three factors, we have identified several relevant observations: (1) BXs are formed by a pair of weakly interacting excitons with rectangular geometry, rather than by a squared BX molecule, as assumed in earlier studies.^37,58^ This is because the strong confinement makes kinetic energy compete against electrostatic one. (2) In thin NPLs, the ionic polarization of the lattice becomes inefficient. This enhances Coulomb attractions and (to a lesser extent) repulsions. (3) Good agreement with experimental BX binding energy (Δ_BX_ ≈ 42 meV) is obtained for monolayered MAPbI3 platelets. (4) The effective dielectric constant felt by BXs approaches ϵ_∞_ for monolayered platelets, but evolves toward ϵ_*s*_ for thicker ones. (5) Haynes rule in 2D holds in these systems, with Δ_BX_/Δ_X_ = 0.10–0.15 (well below the ideal limit of 0.228). The exact value depends on dielectric and lateral confinement. (6) Strengthening dielectric or lateral confinement can double Δ_BX_, with respect to the same NPL with vertical confinement only. The influence of lateral confinement is remarkable because its effect on exciton emission is secondary.

## Supplementary Material



## Notes

Additional calculations, showing the influence of the polaronic radius on the exciton and biexciton binding energies (PDF)

## References

[1] Hintermayr V. A., Richter A. F., Ehrat F., Döblinger M., Vanderlinden W., Sichert J. A., Tong Y., Polavarapu L., Feldmann J., Urban A. S. (2016). Tuning the optical properties of
perovskite nanoplatelets through composition and thickness by ligand-assisted
exfoliation. Adv. Mater..

[2] Weidman M. C., Goodman A. J., Tisdale W. A. (2017). Colloidal
halide perovskite nanoplatelets:
an exciting new class of semiconductor nanomaterials. Chem. Mater..

[3] Otero-Martínez C., Ye J., Sung J., Pastoriza-Santos I., Pérez-Juste J., Xia Z., Rao A., Hoye R. L., Polavarapu L. (2022). Colloidal
metal-halide perovskite nanoplatelets: thickness-controlled synthesis,
properties, and application in light-emitting diodes. Adv. Mater..

[4] Morgenstern T., Lampe C., Naujoks T., Jurow M., Liu Y., Urban A. S., Brütting W. (2020). Elucidating the performance limits
of perovskite nanocrystal light emitting diodes. J. Lumin..

[5] Scott R., Heckmann J., Prudnikau A. V., Antanovich A., Mikhailov A., Owschimikow N., Artemyev M., Climente J. I., Woggon U., Grosse N. B. (2017). Directed emission of
CdSe nanoplatelets originating from strongly anisotropic 2D electronic
structure. Nat. Nanotechnol..

[6] Cho Y., Berkelbach T. C. (2019). Optical
properties of layered hybrid organic-inorganic
halide perovskites: A tight-binding GW-BSE study. J. Phys. Chem. Lett..

[7] Katan C., Mercier N., Even J. (2019). Quantum and dielectric
confinement
effects in lower-dimensional hybrid perovskite semiconductors. Chem. Rev..

[8] Chakraborty R., Nag A. (2021). Dielectric confinement for designing
compositions and optoelectronic
properties of 2D layered hybrid perovskites. Phys. Chem. Chem. Phys..

[9] Jackson, J. D. Classical Electrodynamics; John Wiley & Sons: New York, 1999.

[10] Ithurria S., Tessier M., Mahler B., Lobo R., Dubertret B., Efros A. L. (2011). Colloidal nanoplatelets with two-dimensional electronic
structure. Nat. Mater..

[11] Achtstein A. W., Schliwa A., Prudnikau A., Hardzei M., Artemyev M. V., Thomsen C., Woggon U. (2012). Electronic
structure and exciton-phonon
interaction in two-dimensional colloidal CdSe nanosheets. Nano Lett..

[12] Benchamekh R., Gippius N. A., Even J., Nestoklon M. O., Jancu J.-M., Ithurria S., Dubertret B., Efros A.. L., Voisin P. (2014). Tight-Binding Calculations of Image-Charge
Effects in Colloidal Nanoscale Platelets of CdSe. Phys. Rev. B.

[13] Rajadell F., Climente J. I., Planelles J. (2017). Excitons in
core-only, core-shell
and core-crown CdSe nanoplatelets: Interplay between in-plane electron-hole
correlation, spatial confinement, and dielectric confinement. Phys. Rev. B.

[14] Ferreira A., Létoublon A., Paofai S., Raymond S., Ecolivet C., Rufflé B., Cordier S., Katan C., Saidaminov M. I., Zhumekenov A. A. (2018). Elastic softness of hybrid lead halide perovskites. Phys. Rev. Lett..

[15] Akkerman Q. A., Rainò G., Kovalenko M. V., Manna L. (2018). Genesis, challenges
and opportunities for colloidal lead halide perovskite nanocrystals. Nat. Mater..

[16] Neutzner S., Thouin F., Cortecchia D., Petrozza A., Silva C., Srimath Kandada A. R. (2018). Exciton-polaron
spectral structures in two-dimensional
hybrid lead-halide perovskites. Phys. Rev. Mater..

[17] Zhang L., Hao Y., Wu Y., Qin W., Liu X., Cui B., Xie S. (2020). Self-trapping effect
on the excitonic and polaronic properties of
a single-layer 2D metal-halide perovskite. 2D
Mater..

[18] Guo Y., Yaffe O., Hull T. D., Owen J. S., Reichman D. R., Brus L. E. (2019). Dynamic emission
Stokes shift and liquid-like dielectric
solvation of band edge carriers in lead-halide perovskites. Nat. Commun..

[19] Tao W., Zhang C., Zhou Q., Zhao Y., Zhu H. (2021). Momentarily
trapped exciton polaron in two-dimensional lead halide perovskites. Nat. Commun..

[20] Bao D., Chang Q., Chen B., Chen X., Sun H., Lam Y. M., Zhao D., Zhu J.-X., Chia E. E. (2023). Evidence
of Polaron Formation in Halide Perovskites via Carrier Effective Mass
Measurements. PRX Energy.

[21] Baranowski M., Nowok A., Galkowski K., Dyksik M., Surrente A., Maude D., Zacharias M., Volonakis G., Stranks S. D., Even J. (2024). Polaronic
Mass Enhancement
and Polaronic Excitons in Metal Halide Perovskites. ACS Energy Lett..

[22] Srimath
Kandada A. R., Silva C. (2020). Exciton polarons in two-dimensional
hybrid metal-halide perovskites. J. Phys. Chem.
Lett..

[23] Thouin F., Valverde-Chávez D. A., Quarti C., Cortecchia D., Bargigia I., Beljonne D., Petrozza A., Silva C., Srimath Kandada A. R. (2019). Phonon coherences reveal the polaronic character of
excitons in two-dimensional lead halide perovskites. Nat. Mater..

[24] Gramlich M., Lampe C., Drewniok J., Urban A. S. (2021). How Exciton-Phonon
Coupling Impacts Photoluminescence in Halide Perovskite Nanoplatelets. J. Phys. Chem. Lett..

[25] Baranowski M., Plochocka P. (2020). Excitons in metal-halide perovskites. Adv. Energy Mater..

[26] Yazdani N., Bodnarchuk M., Bertolotti F., Masciocchi N., Fureraj I., Guzelturk B., Cotts B., Zajac M., Rainò G., Jansen M. (2024). Coupling to octahedral
tilts in halide perovskite nanocrystals induces phonon-mediated attractive
interactions between excitons. Nat. Phys..

[27] Menéndez-Proupin E., Beltrán
Ríos C. L., Wahnón P. (2015). Nonhydrogenic
exciton spectrum in perovskite CH_3_NH_3_PbI_3_. pss RRL.

[28] Zhu C., Boehme S. C., Feld L. G., Moskalenko A., Dirin D. N., Mahrt R. F., Stöferle T., Bodnarchuk M. I., Efros A. L., Sercel P. C. (2024). Single-photon
superradiance in individual caesium lead halide quantum dots. Nature.

[29] Movilla J. L., Planelles J., Climente J. I. (2023). Excitons in metal
halide perovskite
nanoplatelets: an effective mass description of polaronic, dielectric
and quantum confinement effects. Nanoscale Adv..

[30] Haken H. (1958). Die theorie
des exzitons im festen Körper. Fortschr.
Phys..

[31] Franceschetti A., Wang L., Fu H., Zunger A. (1998). Short-range versus
long-range electron-hole exchange interactions in semiconductor quantum
dots. Phys. Rev. B.

[32] Ninno D., Trani F., Cantele G., Hameeuw K., Iadonisi G., Degoli E., Ossicini S. (2006). Thomas-Fermi model
of electronic
screening in semiconductor nanocrystals. Europhys.
Lett..

[33] Dyksik M., Wang S., Paritmongkol W., Maude D. K., Tisdale W. A., Baranowski M., Plochocka P. (2021). Tuning the Excitonic Properties of
the 2D (PEA)_2_(MA)_
*n*−1_Pb_
*n*
_I_3*n*+1_ Perovskite
Family via Quantum Confinement. J. Phys. Chem.
Lett..

[34] Sapori D., Kepenekian M., Pedesseau L., Katan C., Even J. (2016). Quantum confinement
and dielectric profiles of colloidal nanoplatelets of halide inorganic
and hybrid organic-inorganic perovskites. Nanoscale.

[35] Macias-Pinilla D. F., Planelles J., Climente J. I. (2022). Biexcitons in CdSe nanoplatelets:
geometry, binding energy and radiative rate. Nanoscale.

[36] Cho Y., Greene S. M., Berkelbach T. C. (2021). Simulations
of trions and biexcitons
in layered hybrid organic-inorganic lead halide perovskites. Phys. Rev. Lett..

[37] Vale B. R., Socie E., Burgos-Caminal A., Bettini J., Schiavon M. A., Moser J.-E. (2020). Exciton, biexciton, and hot exciton dynamics in CsPbBr3
colloidal nanoplatelets. J. Phys. Chem. Lett..

[38] Ishihara T., Takahashi J., Goto T. (1990). Optical properties due to electronic
transitions in two-dimensional semiconductors (C n H 2 n+ 1 NH 3)
2 PbI 4. Phys. Rev. B.

[39] Ishihara T., Hong X., Ding J., Nurmikko A. (1992). Dielectric
confinement
effect for exciton and biexciton states in PbI4-based two-dimensional
semiconductor structures. Surf. Sci..

[40] Kondo T., Azuma T., Yuasa T., Ito R. (1998). Biexciton lasing in
the layered perovskite-type material (C_6_H_13_NH_3_)_2_PbI_4_. Solid
State Commun..

[41] Chong W. K., Thirumal K., Giovanni D., Goh T. W., Liu X., Mathews N., Mhaisalkar S., Sum T. C. (2016). Dominant factors
limiting the optical gain in layered two-dimensional halide perovskite
thin films. Phys. Chem. Chem. Phys..

[42] Thouin F., Neutzner S., Cortecchia D., Dragomir V. A., Soci C., Salim T., Lam Y. M., Leonelli R., Petrozza A., Kandada A. R. S. (2018). Stable biexcitons in two-dimensional metal-halide
perovskites with strong dynamic lattice disorder. Phys. Rev. Mater..

[43] Booker E. P., Price M. B., Budden P. J., Abolins H., del Valle-Inclan
Redondo Y., Eyre L., Nasrallah I., Phillips R. T., Friend R. H., Deschler F., Greenham N. C. (2018). Vertical cavity biexciton lasing in 2D dodecylammonium lead iodide
perovskites. Adv. Opt. Mater..

[44] Villamil
Franco C., Mahler B., Cornaggia C., Gustavsson T., Cassette E. (2021). Auger recombination and multiple
exciton generation in colloidal two-dimensional perovskite nanoplatelets:
Implications for light-emitting devices. ACS
Appl. Nano Mater..

[45] Fang H.-H., Yang J., Adjokatse S., Tekelenburg E., Kamminga M. E., Duim H., Ye J., Blake G. R., Even J., Loi M. A. (2020). Band-edge exciton fine structure
and exciton recombination dynamics in single crystals of layered hybrid
perovskites. Adv. Funct. Mater..

[46] Nagamine G., Rocha J. O., Bonato L. G., Nogueira A. F., Zaharieva Z., Watt A. A., de Brito
Cruz C. H., Padilha L. A. (2018). Two-photon absorption
and two-photon-induced gain in perovskite quantum dots. J. Phys. Chem. Lett..

[47] Cragg G. E., Efros A. L. (2010). Suppression of Auger processes in
confined structures. Nano Lett..

[48] Climente J. I., Movilla J. L., Planelles J. (2012). Auger recombination
suppression in
nanocrystals with asymmetric electron-hole confinement. Small.

[49] Deng S., Shi E., Yuan L., Jin L., Dou L., Huang L. (2020). Long-range
exciton transport and slow annihilation in two-dimensional hybrid
perovskites. Nat. Commun..

[50] Gramlich M., Bohn B. J., Tong Y., Polavarapu L., Feldmann J., Urban A. S. (2020). Thickness-dependence of exciton-exciton
annihilation in halide perovskite nanoplatelets. J. Phys. Chem. Lett..

[51] Li Q., Yang Y., Que W., Lian T. (2019). Size-and morphology-dependent
auger recombination in CsPbBr3 perovskite two-dimensional nanoplatelets
and one-dimensional nanorods. Nano Lett..

[52] Hylleraas E. A., Ore A. (1947). Binding energy of the
positronium molecule. Phys. Rev..

[53] Gramlich M., Swift M. W., Lampe C., Lyons J. L., Döblinger M., Efros A. L., Sercel P. C., Urban A. S. (2022). Dark and bright
excitons in halide perovskite nanoplatelets. Adv. Sci..

[54] Movilla J. L., Planelles J., Climente J. I. (2024). Correction: Excitons in metal halide
perovskite nanoplatelets: an effective mass description of polaronic,
dielectric and quantum confinement effects. Nanoscale Adv..

[55] Blancon J.-C., Stier A. V., Tsai H., Nie W., Stoumpos C. C., Traore B., Pedesseau L., Kepenekian M., Katsutani F., Noe G. (2018). Scaling law for excitons
in 2D perovskite quantum wells. Nat. Commun..

[56] Ceperley D., Chester G. V., Kalos M. H. (1977). Monte Carlo
simulation of a many-fermion
study. Phys. Rev. B.

[57] Planelles J., Climente J. I. (2021). A simple variational quantum Monte
Carlo-effective
mass approach for excitons and trions in quantum dots. Comput. Phys. Commun..

[58] Singh J., Birkedal D., Lyssenko V., Hvam J. M. (1996). Binding energy of
two-dimensional biexcitons. Phys. Rev. B.

[59] Amara M.-R., Said Z., Huo C., Pierret A., Voisin C., Gao W., Xiong Q., Diederichs C. (2023). Spectral fingerprint of quantum confinement
in single CsPbBr3 nanocrystals. Nano Lett..

[60] Zhu C., Feld L. G., Svyrydenko M., Cherniukh I., Dirin D. N., Bodnarchuk M. I., Wood V., Yazdani N., Boehme S. C., Kovalenko M. V., Raino G. (2024). Quantifying
the Size-Dependent Exciton-Phonon Coupling Strength in Single Lead-Halide
Perovskite Quantum Dots. Adv. Opt. Mater..

[61] Lucovsky G., White R., Liang W., Zallen R., Schmid P. (1976). The lattice
polarizability of PbI_2_. Solid State
Commun..

[62] Kleinman D. (1983). Binding energy
of biexcitons and bound excitons in quantum wells. Phys. Rev. B.

[63] Birkedal D., Singh J., Lyssenko V., Erland J., Hvam J. M. (1996). Binding
of quasi-two-dimensional biexcitons. Phys. Rev.
Lett..

[64] Do M., Kim I., Kolaczkowski M. A., Kang J., Kamat G. A., Yuan Z., Barchi N. S., Wang L.-W., Liu Y., Jurow M. J. (2019). Low-dimensional
perovskite nanoplatelet synthesis
using in situ photophysical monitoring to establish controlled growth. Nanoscale.

