# Supplementary material for: Electronic Structure of Biexcitons in Metal Halide Perovskite Nanoplatelets
Source: J Phys Chem Lett. 2024 Jul 12;15(29):7379–86. doi: 10.1021/acs.jpclett.4c01719 (PMC12478872; doi:10.1021/acs.jpclett.4c01719)
Supplement: Supplementary file 1 [file jz4c01719_si_001.pdf]

Supporting Information for "The Electronic Structure of Biexcitons in Metal Halide Perovskite Nanoplatelets"

Juan I. Climente,[*,†] José L. Movilla,[‡] and Josep Planelles[†]

†*Departament de Química Física i Analítica, Universitat Jaume I, E-12080, Castelló de la Plana, Spain*

‡*Dept. d'Educació i Didàctiques Específiques, Universitat Jaume I, 12080, Castelló, Spain*

E-mail: climente@uji.es

In Haken's theory, the contribution of the polar lattice vibrations to the excitonic Hamiltonian is modeled by means of short and long range dielectric screenings, Eq. (2) of the main text. The polaron radius, which is related to the distance where dynamic (electronic) screening evolves into static (electronic plus ionic) one is then given by:[1,2]

$$l_i = \left(\frac{\hbar^2}{2\, m_i\, \hbar\omega_{LO}} \right)^{1/2}, \tag{1}$$

where $i = e, h$ stands for the particle (electron or hole), m_i its effective mass, and $\hbar\omega_{LO}$ the longitudinal optical phonon energy.

In finite nanocrystals, however, surfaces break the translational symmetry and the different bulk optical modes start hybridizing. In lead halide perovskites, this leads to different optical phonon modes (see e.g. Ref.[3]) and to size-dependent exciton-phonon interaction strength (see e.g. Ref.[4]). Furthermore, temperature activates/deactivates some of these modes, and it may introduce additional lattice disorder (see e.g. discussion in Ref.[5]). All these deviations from the ideal conditions prevent a straight identification between the polaron radius and a single phonon mode energy –Eq. (1)–. Rather, the polaron radius should be taken as a phenomenological parameter itself. The electron and hole in-plane polaron radii used in the present work ($l_e \sim l_h \approx 1$ nm) are the same we employed in Ref.,[6] which turned out to reproduce quantitatively the single exciton binding energy measured in $(PEA)_2(MA)_{n-1}Pb_nI_{3n+1}$ Ruddlesden-Popper perovskite layers, with $n = 1, 2, 3$.[7] One can expect them to provide reasonable values for biexcitons in these materials as well.

A systematic view on the influence of the polaron radius on the binding energies is given by Fig. 1. The figure shows Δ_X (top row) and Δ_{XX} (bottom row) for a monolayer ($n = 1$) NPL, where deviations from bulk should be greatest. We assume $l_e = l_h = l$ (this is approximately true in $MAPbI_3$ because $m_e \approx m_h$). The left (right) column shows the results in absence (presence) of dielectric confinement. In all instances, the binding energy increases with the polaron radius, and saturates towards the infinite polaron radius limit. As shown

by Fig. 1(c), in a dielectrically confined NPL, Δ_{BX} increases from 42 to 55 meV when the polaron radius increases from $l \sim 1$ nm to $l \geq 2$ nm. These values are close to the high and low temperature measurements of Ref.[5] The exact numbers should be taken with care, but they offer a qualitative interpretation of the experimental trend. Namely, decreasing the temperature deactivates the optical phonon modes with higher energy. This increases the polaron radius, which scales inversely to the energy of the relevant optical phonons. The net result is an increase in Δ_{BX}.

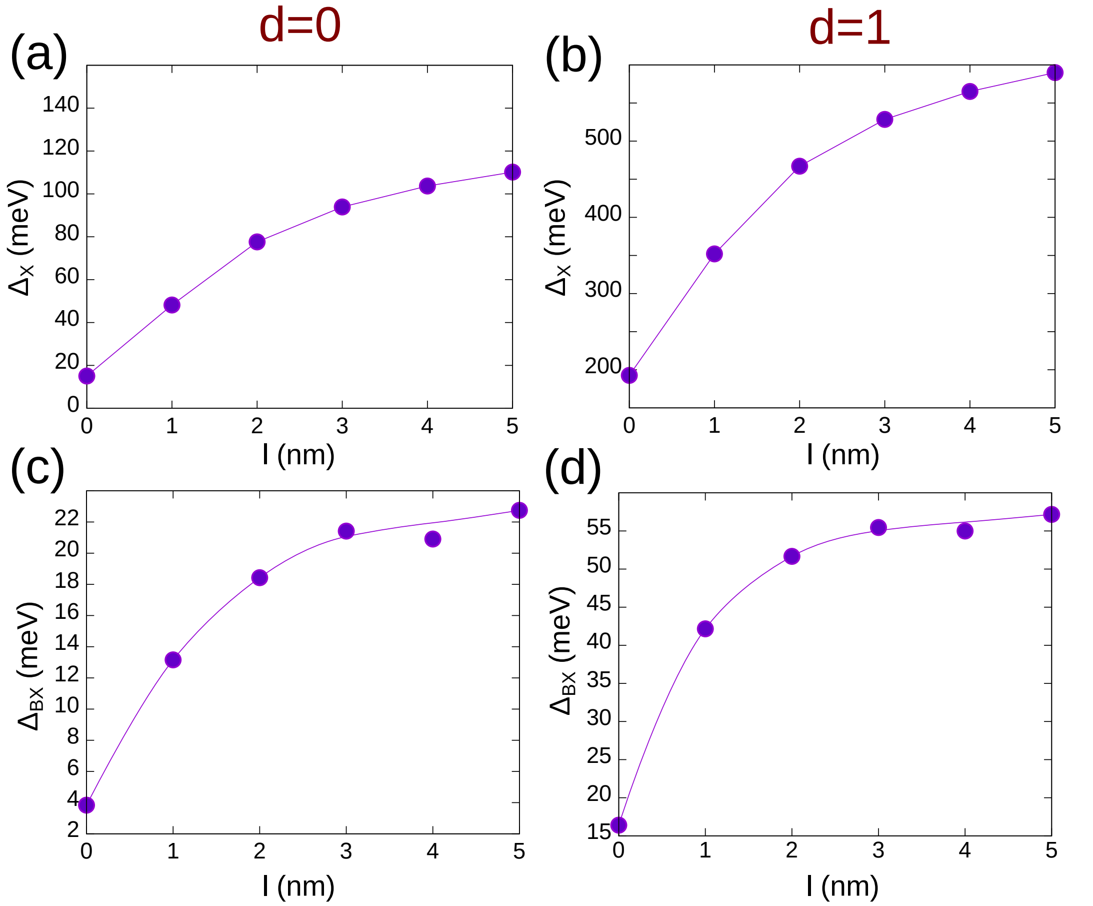

Figure 1: Exciton (a,b) and biexciton (c,d) binding energy as a function of the polaron radius. (a) and (c) correspond to absence of dielectric confinement (no image charges computed). (b) and (d) correspond to presence of dielectric confinement. Dots are calculated points, lines are guides to the eyes.

References

(1) Haken, H. Die theorie des exzitons im festen Körper. *Fortschritte der Physik* **1958**, *6*, 271–334.

(2) Pollmann, J.; Büttner, H. Effective Hamiltonians and bindings energies of Wannier excitons in polar semiconductors. *Physical Review B* **1977**, *16*, 4480.

(3) Amara, M.-R.; Said, Z.; Huo, C.; Pierret, A.; Voisin, C.; Gao, W.; Xiong, Q.; Diederichs, C. Spectral fingerprint of quantum confinement in single CsPbBr3 nanocrystals. *Nano Lett.* **2023**, *23*, 3607–3613.

(4) Zhu, C.; Feld, L. G.; Svyrydenko, M.; Cherniukh, I.; Dirin, D. N.; Bodnarchuk, M. I.; Wood, V.; Yazdani, N.; Boehme, S. C.; Kovalenko, M. V. et al. Quantifying the Size-Dependent Exciton-Phonon Coupling Strength in Single Lead-Halide Perovskite Quantum Dots. *Adv. Opt. Mater.* **2024**, *12*, 2301534.

(5) Thouin, F.; Neutzner, S.; Cortecchia, D.; Dragomir, V. A.; Soci, C.; Salim, T.; Lam, Y. M.; Leonelli, R.; Petrozza, A.; Kandada, A. R. S. et al. Stable biexcitons in two-dimensional metal-halide perovskites with strong dynamic lattice disorder. *Phys. Rev. Mater.* **2018**, *2*, 034001.

(6) Movilla, J. L.; Planelles, J.; Climente, J. I. Correction: Excitons in metal halide perovskite nanoplatelets: an effective mass description of polaronic, dielectric and quantum confinement effects. *Nanoscale Adv.* **2024**, *6*, 2527–2527.

(7) Dyksik, M.; Wang, S.; Paritmongkol, W.; Maude, D. K.; Tisdale, W. A.; Baranowski, M.; Plochocka, P. Tuning the Excitonic Properties of the 2D $(PEA)_2(MA)_{n-1}Pb_nI_{3n+1}$ Perovskite Family via Quantum Confinement. *J. Phys. Chem. Lett.* **2021**, *12*, 1638–1643.
